# Advance of echocardiography in cardiac amyloidosis

**DOI:** 10.1007/s10741-023-10332-3

**Published:** 2023-08-10

**Authors:** Shichu Liang, Zhiyue Liu, Qian Li, Wenfeng He, He Huang

**Affiliations:** Department of Cardiology, West China Hospital, Sichuan University, No. 37 GuoXue Alley, Chengdu, 610041 China

**Keywords:** Cardiac amyloidosis, Echocardiography, Echocardiographic models

## Abstract

Cardiac amyloidosis (CA) occurs when the insoluble fibrils formed by misfolded precursor proteins deposit in cardiac tissues. The early clinical manifestations of CA are not evident, but it is easy to progress to refractory heart failure with an inferior prognosis. Echocardiography is the most commonly adopted non-invasive modality of imaging to visualize cardiac structures and functions, and the preferred modality in the evaluation of patients with cardiac symptoms and suspected CA, which plays a vital role in the diagnosis, prognosis, and long-term management of CA. The present review summarizes the echocardiographic manifestations of CA, new echocardiographic techniques, and the application of multi-parametric echocardiographic models in CA diagnosis.

## Introduction

Cardiac amyloidosis (CA) occurs when the insoluble fibrils formed by misfolded precursor proteins deposit in cardiac tissues [1]. The most common types of CA include immunoglobulin light-chain cardiac amyloidosis (AL-CA) and transthyretin-related cardiac amyloidosis (ATTR-CA), which can be further divided into hereditary ATTR-CA (ATTRm) and wild-type ATTR-CA (ATTRwt or senile ATTR-CA) [2, 3]. While involving the heart, amyloid deposits also affect other organs, such as the spine, kidney, gastrointestinal tract, and nervous system. Such deposits in the heart are prone to progress to refractory heart failure with an extremely poor prognosis. Moreover, due to the non-specific clinical characteristics of CA, half of the patients visited multiple physicians before getting an accurate diagnosis [4], delaying diagnosis and definitive treatment. The mean survival duration for untreated AL-CA patients was less than 6 months [5], and the median survival duration for ATTR-CA patients was only 2.5–3.5 years [6]. Therefore, it is significant to elevate the early clinical identification and detection rates of CA and offer early intervention.

Echocardiography is the most commonly adopted non-invasive modality of imaging to visualize cardiac structure and function. It is also the preferred modality for evaluating patients with cardiac symptoms and suspected CA [7], playing a vital role in the diagnosis, prognosis, and long-term management of CA [8]. In the 2022 ESC Guidelines on cardio-oncology, echocardiography is listed as a class IB recommendation for diagnosing CA [9]. The present review summarizes the advances of echocardiography in the screening and identification of CA.

## Conventional echocardiographic findings of CA

**Fig. 1:**
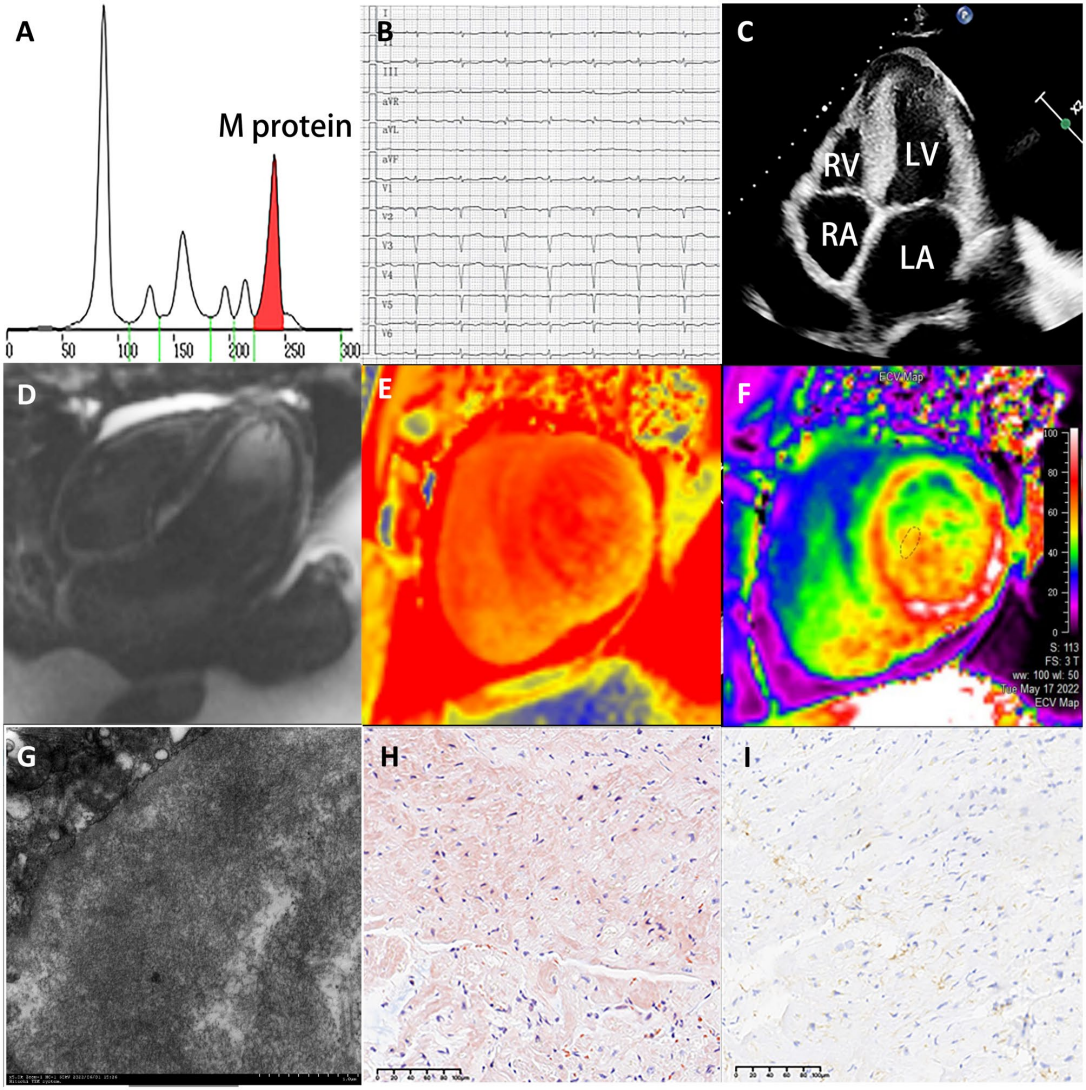
A case presentation of an AL-CA patient. **A** Serum protein electrophoresis showed albumin, α1-globulin, α2-globulin, β1-globulin, β2-globulin, γ-globulin, and M-protein were 39.6%, 5.5%, 18.0%, 5.6%, 6.3%, 25.0%, and 22.5%, respectively. **B** Limb leads electrocardiogram showed low voltage. **C** Echocardiography showed thickening of the LV interventricular septal wall and the LV posterior wall, with “granular” echoes in the interventricular septal wall. **D**–**F** Cardiac magnetic resonance imaging showed subendocardial diffuse late gadolinium enhancement in the 4-chamber view, with an elevated center global native T1 value of 1357 ms in the short-axis view, and an elevated global extracellular volume of 53%. **G** Amyloid fiber deposits were found between the myocardiocyte interstitial and alongside the vessel walls via electron microscopy. **H**, **I** The positive results of Congo red and light-chain stains of myocardial biopsy

**Fig. 2:**
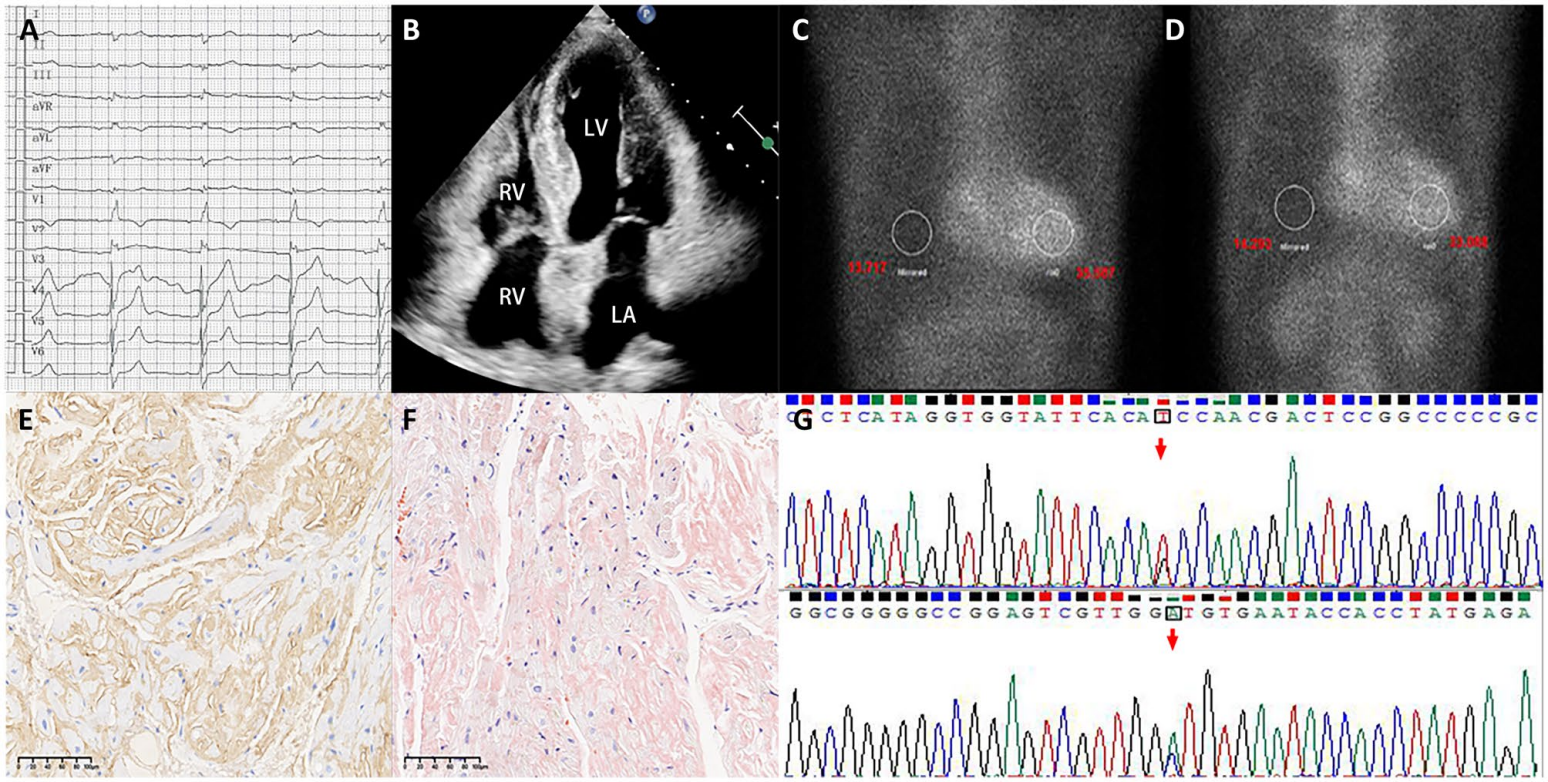
A case presentation of an ATTR-CA patient. **A** Limb leads electrocardiogram showed low voltage. **B** Echocardiography showed asymmetric hypertrophy of the LV interventricular septal wall. **C**, **D**.^99m^Tc-pyrophosphate quantitative SPECT showed that the radiopharmaceutical uptake was more significant in the heart than ribs. The 1-h and 3-h cardiac mean pixel intensities/rib mean pixel intensities were 2.59 and 2.31, respectively. **E**, **F** The positive results of TTR and Congo red stain of myocardial biopsy. **G** The whole exome sequencing showed c.349G > T in chr18:29,178,543

In CA patients, amyloids can deposit in the ventricles, vessels, and valves, leading to wall thickening, left ventricle (LV) volume reduction, biatrial enlargement, and valve thickening. Also, pericardial effusion, pleural effusion, and vena cava dilation are induced by the progression from diastolic dysfunction to restrictive cardiomyopathy [7]. Two typical cases of AL-CA and ATTR-CA are shown in Figs. 1 and 2.

### Ventricular wall hypertrophy

**Fig. 3:**
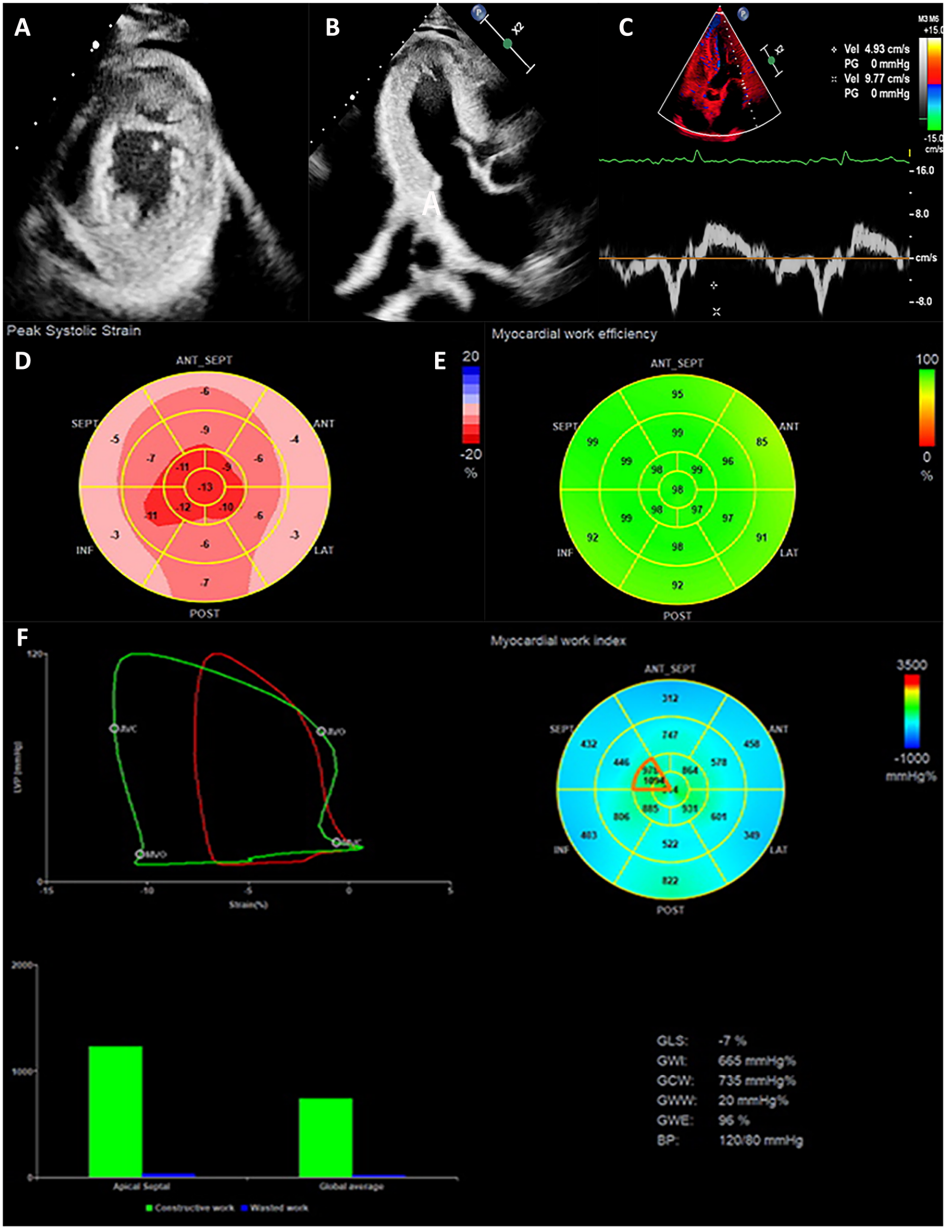
Application of Echocardiography in CA. **A** The apical view of the two-dimensional echocardiography showed the ventricular hypertrophy. **B** The three-chamber view of the two-dimensional echocardiography showed the hypertrophy and “granular” of the LV interventricular septal wall. **C** The tissue Doppler showed a decreased mitral annular motion. **D** The two-dimensional speckle-tracking echocardiography showed apical sparing of the LV segmental longitudinal strain. **E**, **F** LV pressure-strain myocardial work showed an apical sparing of the LV segmental myocardial work and myocardial efficiency

LV hypertrophy (LVH) is CA patients’ most common echocardiographic finding (Fig. 3A) [10]. It was reported that with the help of gene detection, nearly 5% of the patients initially diagnosed with hypertrophic cardiomyopathies were eventually diagnosed as ATTRm [11].

The ventricular wall is the most commonly involved by amyloid deposition, which can result in non-dilated ventricular hypertrophy, thus leading to decreased ventricular volume. Such ventricular hypertrophy is usually symmetrical in AL-CA patients but asymmetrical in ATTR-CA patients [7]. Different from the LVH caused by other factors, the electrocardiography (ECG) features of CA-induced LVH (CA-LVH) are inconsistent with the echocardiographic findings of LVH. It is mainly characterized on the ECG by low voltage in limb leads, poor R-wave progression in the right chest lead, and significantly reduced R-wave voltage in V5 and V6 leads. LVH of unspecific causes and the low voltage pattern suggest CA. However, these are not the characteristic manifestations of CA, with the ECG low-voltage pattern only observed in 40–76% of CA patients [12]. In the absence of aortic valve diseases or under severe hypertension, LV thickness > 12 mm and diastolic dysfunction of grade II or above are highly indicative of CA [13]. Nevertheless, normal LV thickness can be witnessed in about one-third of the CA patients, which means LVH is not necessarily associated with the degree of amyloid deposition. CA cannot be completely ruled out in patients without LVH [14].

In addition, amyloid deposits of CA can not only involve LV, but also implicate the right ventricle (RV), the atria, and atrial septum. If the RV is affected, the thickness of the RV wall might be greater than 5 mm. If the atria are involved, the atrial septum (> 5 mm) and atrioventricular valve (> 2 mm) become thickened [15, 16]. A study showed that an atrial septal thickness greater than 6 mm was 100% specific for diagnosing CA [17].

### Biatrial enlargement

The involvement of amyloids in both ventricles leads to ventricular wall hypertrophy and diminished diastolic functions, which will further lift the ventricular pressure, and ultimately result in biatrial enlargement and atrial wall hypertrophy. Enlarged left atria (LA) can reflect the severity and duration of diastolic cardiac dysfunction, which can serve as the imaging marker of the early subclinical changes of ATTR-CA [18]. Besides, LA enlargement is also a high-risk factor for thrombosis. Marked thrombosis can be observed in some CA patients with enlarged LA, potentially associated with endocardial disturbances, atrial remodeling, diastolic dysfunction, and a hypercoagulable state [19]. Meanwhile, the increased LA filling pressure can also give rise to right atria (RA) pressure, characterized by the inferior vena cava’s widened internal diameter and reduced respiration collapse rate [20].

### Valve thickening

Aortic stenosis (AS) and CA are two different disease processes, but there are multiple common risk factors, so the overlap between AS and CA is rather usual. It was estimated that about 15% of AS patients also suffered from CA [16]. In CA patients, 16% of the ATTRwt patients [21] and 9% of the AL-CA patients [22] also had AS. In most cases, degenerative AS is caused by the hypertrophy and calcification of valve leaflets due to proliferative and inflammatory lesions. However, amyloid deposition can facilitate this progression, leading to LVH, cardiac diastolic, and systolic dysfunction, eventually resulting in heart failure [23]. In AS assessment, CA patients may present as low-flow and low-gradient AS with preserved LV ejection fraction (LVEF), i.e., LVEF > 50%, transvalvular pressure < 40 mmHg, valve orifice area < 1 cm^2^, and stroke volume index < 35 ml/m^2^. It was expected that about 30% of the low-flow and low-gradient AS patients with preserved LVEF might also have CA [16]. Peskó et al. [22] performed dobutamine stress echocardiography (DSE) in 3 patients with severe low-flow and low-gradient AS and identified one patient with true-severe AS and two with pseudo-severe AS. DSE might be able to assess the severity and characteristics of AS in CA patients.

Apart from thickening and stenosis of the aortic valve, CA patients may also present with thickening of the mitral and tricuspid valves, which leads to mitral and tricuspid regurgitation of different degrees [24].

### Heart failure with preserved ejection fraction in early stage

Patients with early CA exhibit no evident decrease in LVEF but may undergo unexplained symptoms such as progressive heart failure and dyspnoea, indicative of heart failure with preserved ejection fraction (HFpEF). CA is considered one of the neglected etiologies of HFpEF in the elderly [25]. The myocardial deformation featuring the accumulation of amyloids is in a dual-gradient (basal-to-apical and subendocardium-to-subepicardial) pattern, in which subendocardial myocardial fibers are responsible for the longitudinal deformation. In contrast, subepicardial myocardial fibers often involve circumferential deformation [26]. The spiral arrangement enables the myocardium to contract more under increased LV end-diastolic pressure (LVEDP) to retain ventricular functions. The declined LVEF in late-stage CA patients may be associated with myocardial torsional decompensation [27]. Patients with declined LVEF present as heart failure with reduced ejection fraction (HFrEF), which might be an echographic marker for more severe disease [6].

### Restrictive diastolic dysfunction

Compared with systolic dysfunction, restrictive diastolic dysfunction is induced by amyloid involvement in myocardial tissues. It is also more prominent, which is often of grade II or above, accompanied by an increased ratio of early diastolic mitral flow velocity to late diastolic mitral flow velocity (E/A, > 1.5) and a shortened duration of E-wave deceleration (< 150 ms) [13]. The LV filling pressure increases when the ratio of early diastolic mitral flow velocity to mitral annular motion velocity (E/e’) > 14. Previous studies revealed a significant increase of E/e’ (approximately 18) in CA patients unrelated to LVH [14, 28]. In addition, the tissue Doppler imaging of the mitral annulus exhibited a “5–5-5 pattern”, i.e., the e’-, a’-, and s’-waves all < 5 cm/s (Fig. 3C) [12], which is highly suggestive of CA but may not be evident in the early stage of the disease [13]. The restrictive diastolic dysfunction caused by various types of CA may be different. More specifically, AL-CA may lead to restrictive diastolic dysfunction in the early disease stage, accompanied by mild or no ventricular wall thickening, which will subside through effective chemotherapy. Nevertheless, ATTR-CA may be associated with restrictive diastolic dysfunction in the mid-to-late stage of the disease [29]. However, it should be noted that CA cannot be diagnosed through the restrictive filling pattern detected by only one echocardiographic examination because transient hemodynamic presentations may cause such pathophysiological manifestations. For this reason, a restricted filling pattern reviewed by echocardiography at least twice (with the interval being at least 6 months) can be considered a “persistent” restricted pathophysiological manifestation [29].

### Myocardial echogenicity

Amyloid deposition can also bring out “sparkling” or “granular” echoes or “ground-glass” enhancement in 25% of CA patients (Fig. 3B) [30]. However, it has been proved to be only a non-specific presentation that can be found in patients with end-stage renal diseases and other infiltrative cardiomyopathies [31].

### Pericardial effusion

The accumulation of amyloids in the pericardial cavity leads to pericardial effusion. CA patients primarily present with a tiny or small amount of pericardial effusion, while some patients may suffer from chronic pericardial effusion [32] that repeatedly recurs [33]. The correlation between pericardial effusion and right cardiac insufficiency is still ambiguous [34]. However, pericardial effusion enables the risk stratification of CA [6].

If patients meet the characteristics above, CA should be highly suspected. The subsequent step examination, such as cardiac magnetic resonance (CMR) imaging, ^99m^technetium-pyrophosphate (^99^Tc^m^-PYP) single-photon emission computed tomography (SPECT), and cardiac biopsy should be taken. Kanelidis et al. [35] reported a patient with IgG κ light-chain multiple myeloma (MM) with manifestations such as heart failure during chemotherapy. Due to the high level of the κ light chain, the patient was initially suspected of having AL-CA but was eventually diagnosed with ATTR-CA through the proteomic analysis of biopsied tissues by liquid chromatography-tandem mass spectrometry, and treated with tafamidis soon. The case indicated that though echocardiography is of vital importance in the screening of CA, cardiac biopsy can accurately diagnose CA and its subtypes.

## Application of new techniques in CA

Speckle-tracking echocardiography, myocardial work and myocardial contraction fraction are new echocardiographic techniques in CA. These techniques can be used in the possible diagnosis and prognosis prediction of CA (Fig. 3 and Table 1).

**Table 1:** Application of new techniques in CA

**Index**	**CA possibility**	**Sensitivity**	**Sensitivity**	**Reference**
**Possible diagnosis of CA**				
**Apical sparing**	Possible CA	93%	82%	[40]
**LVEF/GLS > 4.95**	Possible CA	75%	66%	[44]
**GLS ≤ 16.10%**	Possible CA	92.9%	93.7%	[45]
**GAS ≤ 32.95%**	Possible CA	81%	53.1%	
**GLS ≤ 16.09%**	Possible CA in AL	94.23%	87.5%	[46]
**GAS ≤ 36.54%**	Possible CA in AL	86.54%	80%	
**GRS ≤ 31.90%**	Possible CA in AL	80.8%	47.5	
**GAS < 19.4%**	Possible CA	67.70%	75%	[47]
**RV apical ratios > 0.8**	Differentiating AL-CA and ATTR-CA	97.80%	90%	[51]
**GWE < 86.5%**	Differentiating AL-CA and ATTR-CA	80%	66.7%	[57]
**Poor prognosis of CA**				
**GAS < −19%**	HR = 1.23	–	–	[47]
**Basal longitudinal strain ≤ 13.07%**	HR = 0.812 (0.675–0.976)	–	–	[48]
**LVMWI < 1039 mmHg%**	HR = 6.4 (2.4–17.1)	–	–	[58]
**LVMWE < 89%**	AUC = 0.689 (0.597–0.771)	65%	48%	[60]
**MCF < 25%**	HR = 5.369 (2.4–1.817–15.86)	–	–	[62]

*AL-CA* light-chain cardiac amyloidosis, *ATTR-CA* transthyretin-related cardiac amyloidosis, *CA* cardiac amyloidosis, *GLS* global longitudinal strain, *GAS* global area strain, *GRS* global radical strain, *GWE* global work efficiency, *LVEF* left ventricular ejection fraction, *LVMWI* left ventricular myocardial work index, *LVMWE* left ventricular myocardial work efficiency, *MCF* myocardial contraction fraction

### Speckle-tracking echocardiography

Amyloids can involve multiple cardiac chambers. Kado et al. [36] indicated that the longitudinal strain (LS) measured from the four-chamber view reflected the overall load of cardiac function. The study also pointed out that the four-chamber LS of CA-LVH patients was lower than that of the average population and was associated with the major adverse cardiovascular events of CA patients. The LV global longitudinal strain (GLS), superior to conventional echocardiographic indicators, is an early sensitive indicator evaluating the subclinical changes in left cardiac function, helping predict the prognosis of the heart disease that results in LVH [37]. CA patients may exhibit apical sparing, characterized by decreased LV-GLS with reduced LS in the basal and mid segments and nonsignificantly decreased LS in the apical segment (Fig. 3D) [38, 39]. The phenomenon of apical sparing suggests that more amyloids might accumulate at the heart base. Ternacle et al. [26] demonstrated that apical LS > −14.5% indicated severe heart involvement and was an independent risk factor for predicting adverse cardiovascular events. Despite the high sensitivity (93%) and specificity (82%) of apical sparing in distinguishing CA-LVH from LVH induced by other factors [40], it is not a sign specific to CA, which can also occur in patients with other infiltrative heart diseases such as Danon disease [41], Fabry disease [42], and end-stage kidney diseases [43]. Kyrouac et al. [44] considered LVEF/GLS a better tool for CA screening, with a sensitivity of 75% and specificity of 66% when LVEF/GLS > 4.95.

Based on GLS and global circumferential strain (GCS), global area strain (GAS), obtained from three-dimensional (3D) speckle-tracking echocardiography, is a new comprehensive indicator reflecting the myocardial motion in all directions. Lei et al. [45] conducted 3D speckle-tracking echocardiography to measure the echocardiographic indicators of CA patients. Also, it indicated that baseline GLS ≤ 16.10% and GAS ≤ 32.95% implied cardiac involvement among patients with systemic amyloidosis, a sensitivity and specificity of 92.9% and 93.7% for GLS, and 81% and 53.1% for GAS, respectively. Lei et al. [46] also reached a similar conclusion that AL patients with GLS ≤ 16.09%, GAS ≤ 36.54%, and global radical strain (GRS) ≤ 31.90% were more prone to cardiac involvement. Besides, GAS < −19% [47] and basal LS ≤ 13.07% [48] can serve as prognostic factors among CA patients.

RV involvement is also common in the early involvement of CA patients. Among them, RV-LS can lead to apical sparing, similar to the phenomenon associated with LV-GLS [49, 50]. Moñivas et al. [51] discovered that AL-CA patients had higher RV apical/(basal + middle) ratios than ATTR-CA patients. Also, when the ratio was greater than 0.80, it could provide a reference basis for differentiating CA subtypes. In distinguishing AL-CA from ATTR-CA, the sensitivity, specificity, and accuracy reached 97.8%, 90.0%, and 94.7%.

However, the manifestations differ slightly between CA patients with LA and RA involvement. CA patients with impaired LA primarily present with the weakened pump and reservoir function, while the conduit function becomes a compensatory mechanism [52]. LA-LS is independently associated with the prognosis of CA patients, and LA-LS combined with RV-free wall strain is highly valuable in determining the prognosis of CA patients [53]. Compared with the average population, CA patients with RA-LS present with declined pump, reservoir, and conduit functions, among which the RA reservoir and conduit functions are correlated with the prognosis of CA patients and can be the potential myocardial markers in the risk stratification of CA [54].

### Myocardial work

Based on the measurement of myocardial strain, myocardial work is a parameter that considers the effects of LV deformation and afterload during the assessment of LV pressure, providing references for the evaluation of cardiac function (Fig. 3E, F) [55]. The LV pressure-strain loop, integrating the measurement of myocardial strain and LV pressure, is a standard evaluation method of myocardial work. It can better reflect the early changes in LV systolic functions among HFpEF patients with different diastolic functions than GLS. Clemmensen et al. [56] revealed that CA patients’ LV myocardial work index (LVMWI) was significantly lower than the average population, which could increase through exercise. However, the constantly declining LV myocardial work efficiency (LVMWE) suggested the low utilization rate of myocardial energy in CA patients. By assessing the cardiac load of patients with CA and HFpEF through LVMWE, Palmiero et al. [57] found that the global work efficiency (GWE) was lower in AL-CA patients compared with ATTR-CA patients, implying that the myocardial dysfunction in AL-CA patients was more evident. They also discovered that AL-CA and ATTR-CA could be appropriately distinguished when GWE < 86.5%, with a sensitivity and specificity of 80.0% and 66.7%, respectively [57]. When LVMWI < 1039 mmHg%, the risk of all-cause mortality in CA patients visibly increased with a hazard rate of 6.4 (95%CI: 2.4–17.1) [58]. Low LVMWE is the potential predictor of adverse cardiovascular events in CA patients [59], and patients with LVMWE < 89% are faced with a high risk of all-cause mortality [60].

### Myocardial contraction fraction

Myocardial contraction fraction (MCF) is the ratio of stroke volume/LV volume that can be adopted to evaluate the myocardial contraction capacity of CA patients. MCF can differentiate CA-LVH from LVH caused by other factors. The MCFs are 60–75% in patients with physiological hypertrophy, such as athletes, 30–45% in patients with HFpEF induced by systemic inflammation or metabolic diseases, 35–45% in patients with hypertrophic cardiomyopathies, 20–30% in CA patients, and 15–40% in heart failure patients with reduced LVEF [61]. Patients with lower than 25% MCF have a higher risk of death [62].

## Application of multi-parametric echocardiographic models

Although the diagnosis of CA cannot be confirmed via echocardiography, some seemingly non-specific signs combined are highly suggestive of CA. Therefore, multi-parametric echocardiographic models can facilitate CA diagnosis, as shown in Table 2.

**Table 2:** Multi-parametric echocardiographic models in CA

	**Population**		**Entries**	**Points**	**Scores**	**CA possibility**	**Sensitivity**	**Sensitivity**	**Reference**
	**CA group**	**Non-CA group**							
**AL score**	332 systemic	172 systemic	RWT > 0.52	2	< 1	Unlikely	100%	0%	
***AUC* = 0.90**	AL	AL	E/e’ > 10	2	1–4	Possible	93%	43%	[65]
**(95%Cl:0.87–**	amyloidosis	amyloidosis	TAPSE ≤ 19 mm	1	≥ 5	Highly likely	54%	98%	
**–0.92)**	with CA	without CA	LS ≤ −14%	1					
**IWT score**			RWT > 0.6	3					
***AUC* = 0.87**			Ele’ > 11	1	< 2	Unlikely	98%	19%	
**(95%Cl: 0.85–**	647 with LVH	331 with LVH	TAPSE ≤ 19 mm	2	2–7	Possible	61%	27%	[65]
**0.90)**	and	but without	LS ≤ −13%	1	≥ 8	Highly likely	46%	98%	
	CA	CA	SAB > 2.9	3					
**ATTR-CM**			Age 60–69	2					
**score**			Age 70–79	3					
***AUC* = 0.89**	189 ATTR-CA	227 HFpEF	Age ≥ 80	4	≥ 6	Highly likely	83%	72%	[66]
**(95%Cl: 0.86–**			Male	2					
**0.92)**			LVEF < 60%	−1					
			PWT ≥ 12 mm	1					
**AMYLI core**	67 AL-CA	184 unexplained LVH	RWT × E/e’	–	< 2.2	Unlikely	100%	5%	[67]
***AUC* = 0.79**					> 26.79	Highly likely	4%	99%	
**Nakao et al.**			Age (men ≥ 65; woman ≥ 70)	1					
***AUC* = 0.88**	54 CA	241 LVH	Low ECG Voltage	1	≥ 2	Highly likely	71%	93%	[68]
**(95%Cl: 0.82**			PWT ≥ 14 mms	1					
**–0.93)**			RASP	1					
**Nicol et al.**	82 systemic	32 systemic	Hs-troponin T > 35 ng/L	1					
***AUC* = 0.97**	AL amyloidosis	AL amyloidosis	GLS ≥ −17%	1	> 1	Possible	94%	97%	[69]
**(95%Cl: 0.90–0.99)**	with CA	without CA	Apical sparing of GLS ≥ 0.90	1					

*AL-CA* light-chain cardiac amyloidosis, *ATTR-CA* transthyretin-related cardiac amyloidosis, *CA* cardiac amyloidosis, *GLS* global longitudinal strain, *HFpEF* heart failure with preserved ejection fraction, *LVEF* left ventricular ejection fraction, *LVH* left ventricular hypertrophy, *PWT* posterior wall thickness, *RWT* relative wall thickness, *RASP* relative apical sparing patterns of longitudinal strain

### Conventional echocardiographic models

Wang et al. [63] compared the conventional echocardiographic parameters and strains of patients with CA to those with hypertensive LVH. They included LV end-diastolic dimension (LVEDD), LVEF, A peak, enhancement of myocardial echogenicity, RV wall thickness, GLS, and apical sparing in their new model, the area under the curve (AUC), and sensitivity. Also, the specificity of distinguishing patients with CA and hypertensive LVH was 0.957, 91.89%, and 94.74%, respectively. In addition, it was reported by Pagourelias et al. [64] that LVEF/GLS presented with favorable sensitivity (89.7%) and specificity (91.7%) in identifying CA with LVEF and mild LVH. However, due to the low incidence of CA, the number of cases included in most studies is currently limited, and further explorations with expanded cohort sizes are still required.

### AL and IWT scores

Boldrini et al. [65] established the scoring systems of AL and increased wall thickness (IWT) based on the multiple echocardiographic parameters among a large cohort. The AUCs of AL and IWT scores were found to be 0.90 and 0.87, respectively. CA could be excluded when the AL score was < 1 or the IWT score was < 2 and diagnosed when the AL score was ≥ 5 or the IWT score was ≥ 8. Therefore, the two scoring systems can confirm or exclude the diagnosis of half of the patients with systemic AL or those with suspected ventricular wall hypertrophy. The scores are positively correlated with the content of myocardial amyloid proteins.

### ATTR-CM score

Davies et al. [66] created the ATTR-CM scoring system to screen for high-risk ATTR-CA patients among the HFpEF group and found that patients with > 6 points were undergoing high-risk CA with a positive predictive value (PPV) of ≥ 25%.

### Other scores

Aimo et al. [67] selected the parameters of relative wall thickness (RWT) and E/e’ included by both AL and IWT scoring systems to set up the AMYLI scoring system (RWT × E/e’). They discovered that when the AMYLI score < 2.36 and < 2.22, suspected AL-CA and unexplained ventricular hypertrophy could be ruled out. Evidence was provided by Nakao et al. [68] to prove the value of apical sparing in CA diagnosis. Through multivariate analysis, the study finally worked out four scoring criteria: 1 point for males aged > 65 years (females aged > 70 years), ECG low voltage in limb leads, LV posterior wall thickness ≥ 14 mm, and presence of apical sparing. Over 60% of the subjects with 2 points were diagnosed with CA, and the figure increased to 85% among patients with ≥ 3 points. Nicol et al. [69] found that Hs troponin T > 35 ng/L, combing with GLS ≥ −17% and apical sparing of GLS ≥ 0.90, can accurately detect cardiac involvement in AL amyloid patients.

### Artificial intelligence models

The acquisition and identification of echocardiographic images and values mainly rely on the experience and subjective judgment of operators, and sometimes, it is easy to omit tiny changes in a few cases. Comparatively, artificial intelligence (AI) can push the limits of the spatial resolution of human eyes, identify pixels accurately, and utilize image information comprehensively. Also, AI conducts sophisticated quantitative analysis automatically, thereby diminishing human intervention and realizing standardized echocardiographic monitoring [70]. The AI-based echocardiographic texture analysis can be applied to identify the etiologies of LVH [71]. Goto et al. [72] established an AI model based on multi-center CA patients’ ECG and echocardiographic findings. The AUC of the echocardiography was 0.89–0.91, and the adoption of ECG pre-screening elevated the recall rate of the echocardiographic model by 67% and lifted the positive predictive value from 33 to 74–77%. Besides, Yu et al. [73] created a semi-automatic diagnostic network by comparing the echocardiograms of hypertensive heart disease, or hypertrophic cardiomyopathy. In addition, CA with the deep-learning algorithm showed that the LVH detection model could identify the etiologies of LVH with the AUC, sensitivity, and specificity of 0.98, 94%, and 91.6%, respectively.

## Conclusions

Echocardiography, the most used detection method of cardiac structures and functions, is vital in the early screening and diagnosis of CA. With new techniques and multi-parameter scores, echocardiography can play a better role in the diagnosis and prognosis of CA.

## Data Availability

Not applicable.
